# Impressions

**Published:** 1860-01

**Authors:** M. M. Oldham

**Affiliations:** Springfield, Ohio


### IMPRESSIONS.

BY DR. M. M. OLDHAM, SPRINGFIELD, OHIO.

Read at a meeting of the Mad River Valley Dental Association.

GENTLEMEN: I have a very common place subject, hence you need not expect anything more than common place remarks on it: yet to the practical dentist, it is a subject of importance, and holds a similar place in mechanical dentistry, that the corner stone and its fellows do to the superstructure. Unless you have a correct impression of the mouth, or of such parts of it as are to be covered by the plate, your artificial denture however skillfully made, and neatly finished it may be, will be found to be altogether worthless for any practical purpose in the mouth; for it can not be worn with comfort, or utility. It is therefore important that a correct beginning be made in every operation intended for the mouth.

Taking an impression of the mouth is a simple operation, yet one requiring the exercise of some care and skill, in order to be properly and accurately done. There are several things to be observed previous to taking an impression in order to make it a success. The first thing to be observed is the condition of the mouth, if slimy mucus covers the mouth, have the patient rinse it thoroughly with cold water; or if the lining membranes of the cavities of the mouth are thick, and yield ready to pressure on any part so that it may be changed by an impression, greater accuracy may be secured to it, by frequently rinsing the mouth with cold water for a minute or two, which will stimulate the tissues covering the palatal bones, to contract for the time, and thus afford a more unyielding surface.

The next thing is the selection of a proper vessel or cup to hold the material with which the impression is to be taken. This cup should be as closely adapted to the mouth as is convenient, so that as small amount of material, (and especially if it is wax,) as possible may be used, for if a considerable quantity is used, it will be cumbersome to the patient, and likely to produce gagging and consequently violent contraction of the muscles of the throat and face, and thus defeat the success of the operation. Again, if too much wax is employed, it will be forced out at the posterior part of the cup, giving the surface of the impression the appearance of lines or ridges which have no correspondence in the mouth, and is likewise liable to be pulled up or thickened, thus spoiling the correctness of the impression. The cup should also be sufficiently strong to bear the force without springing—to give a perfect adaptation of the wax to all parts of the mouth to be operated upon.

And further, much depends on the material employed and its preparation, in order to secure a correct impression. In all ordinary cases we think wax is preferable, on account of its convenience and pleasantness to the patients. If it is resolved to use wax it should be of the best quality of yellow bees-wax; we think the yellow preferable to the white, because it contains all its natural glutinous properties, so that when heated to a plastic state, it changes the shape of its surface with a smoothness, which adapts it very well for taking impressions with much accuracy. In preparing wax for an impression, much care should be exercised to bring it to the required plasticity, which may be done either by submerging it in warm water or exposing it to the heat of a spirit-lamp, taking care not to overheat the surface so as to destroy the tenacity, or render brittle and lumpy, for with wax in this condition a good and correct impression can not be obtained, for it will not readily adapt itself to the parts of the mouth as desired.

The temperature of the wax should be gradually raised, at the same time working or kneading it with the fingers, till the heat is regularly diffused through the whole mass, and it is rendered to as plastic a condition as desirable, which, if then properly applied to the mouth, can not fail to give in return a perfectly reliable impression.

If you resolve to take the impression with plaster, let it be of the best quality, such as will harden quickly; if good it needs no other preparation to fit it for use but to add as much water as will add to the consistency of thick cream.

The mouth being in a proper condition, and a suitable cup at hand, and the wax in a proper state of preperation, insert as much of the wax as is necessary to cover the alveolar ridge, and such parts as it is desirable to obtain a model of, molding it (the wax) with the fingers, to as nearly the proper shape as possible, then grasping the cup with the right hand, holding the handle firmly between the fore finger and thumb; with the left hand introduce a pearl or ivory spatula to clear the lips and cheeks out of the way of the cup. Insert the cup into the mouth, placing it as fairly over the ridge as possible; then with the first two fingers of the right hand, and with the first one of the left placed on the back of the cup, make firm and steady pressure till the wax has adapted itself to all parts. Allow the wax to cool and then remove with care from the mouth. We may add here, if taking an impression for a section plate, it will be well to have at hand some cold water and a sponge, so that when the impression is taken as above described, apply the sponge saturated with water, to the back of the cup to cool the wax, so that on removing it from the mouth it will prevent it from being pressed out of shape and thus spoiling the impression; and for further security in removing the impression from the mouth, it will be necessary to make an opening through the wax to the palate, so as readily to admit air between the wax and palate, and at the same time on attempting to remove it, to turn up the lip with a spatula or the finger so as to admit air at the border. With these precautions and a little address on the part of the operator, the impression may be readily removed successfully. These things observed, we seldom fail to get a good impression of such parts of the mouth as is desired.

There may however cases present themselves, in which it is impossible to get a correct impression with wax; in such, we must resort to the use of plaster. These cases are such as have soft places on the ridge or palates which yield so readily to the force employed in taking a wax impression that the natural form of the parts is not transferred. The use of plaster in such cases is preferable, because it can be employed in a batter like state, so as to need the application of but little force to completely embrace the desired parts in it, and from this a model can be obtained which will be a fac simile of such parts and a perfect adaptation of a plate to the mouth is secured.

